# Impact of sputum neutrophilia on the efficacy of biologics in severe asthma

**DOI:** 10.3389/falgy.2026.1789783

**Published:** 2026-04-08

**Authors:** Patrizia Pignatti, Martina Zappa, Francesco Ardesi, Marco Vanetti, Rosella Centis, Antonio Spanevello, Dina Visca

**Affiliations:** 1 Allergy and Immunology Unit, Istituti Clinici Scientifici Maugeri IRCCS, Pavia, Italy; 2 Department of Medicine and Cardiopulmonary Rehabilitation, Istituti Clinici Scientifici Maugeri IRCCS, Tradate, Italy; 3 Department of Medicine and Surgery, University of Insubria, Varese, Italy; 4 Servizio di Epidemiologia e Clinica delle Malattie Respiratorie, Istituti Clinici Scientifici Maugeri IRCCS, Tradate, Italy

**Keywords:** airway inflammation, eosinophils, lung function, monoclonal antibodies, neutrophils

## Abstract

**Introduction:**

Patients with severe asthma have new therapeutic opportunities with biologic agents, reducing exacerbation rates, symptom scores, and oral corticosteroid use; however, their effects on lung function appear to be variable. The aim of this study is to evaluate the clinical and inflammatory outcomes of biologic therapy in patients with severe asthma, stratified according to baseline bronchial inflammation.

**Methods:**

This was a retrospective observational study in patients with severe asthma at 6 and 12 months after initiation of biologic therapy. Patients were categorized according to their baseline airway inflammatory profile. The inflammatory biomarkers evaluated included induced sputum, fractional exhaled nitric oxide (FeNO), and peripheral blood leukocyte counts. Lung function, comorbidities, exacerbation rate, and asthma control (assessed by ACQ-6 and ACT) were also recorded.

**Results:**

A total of 113 patients with severe asthma were analyzed. Patients with a paucigranulocytic pattern were excluded from further analyses due to their small number (*n* = 10). Among the remaining subjects (*n* = 103), 62.8% exhibited an eosinophilic pattern, 13.3% a mixed granulocytic pattern, and 15.0% a neutrophilic pattern. Most neutrophilic patients (82.7%) presented elevated type 2 (T2) biomarkers (FeNO and/or blood eosinophils). Differences in baseline biomarkers and comorbidities reflected the underlying airway inflammatory patterns; forced vital capacity (FVC, L) was lower in neutrophilic patients compared with the other groups. Neutrophilic patients had higher frequencies of obstructive sleep apnea and lower chronic rhinosinusitis with nasal polyps than eosinophilic subjects. During follow-up, all patients showed a significant reduction in their ability to produce sputum (*p* < 0.001), as well as significant decreases in exacerbation rate and symptom burden. Eosinophilic and mixed granulocytic patients exhibited significant improvements in lung function, whereas neutrophilic patients did not. Only eosinophilic patients showed a significant reduction in airway inflammation. Oral corticosteroid doses decreased across all groups, but significantly only in eosinophilic patients. Clinical and inflammatory improvements were observed after 6 months of biologic therapy, with no further significant changes at 12 months.

**Conclusions:**

After 12 months of biologic therapy, patients showed phenotype-dependent responses, with neutrophilic patients demonstrating smaller clinical and inflammatory improvements compared with those with eosinophilic or mixed granulocytic phenotypes.

## 1 Introduction

In recent years, patients with severe asthma have gained access to new therapeutic options aimed at achieving better disease control (1). Monoclonal antibodies targeting specific molecules implicated as key drivers of the disease have been introduced and approved as add-on treatments to inhaled corticosteroids and long-acting β2-agonists (ICS/LABA) in patients with severe asthma. The first monoclonal antibody approved several years ago was an anti-IgE, indicated for allergic patients in whom the primary disease driver is clearly attributed to an atopic profile, while most significant predictors of response seem to be breathomics and plasma lipidomics biomarkers (2). Subsequently, interleukin-5 (IL-5) was identified as a key pathogenic driver in a subset of patients with severe asthma, particularly those with late-onset disease and an eosinophilic pattern of systemic and/or airway inflammation. Therefore, monoclonal antibodies targeting IL-5 (mepolizumab, reslizumab) or its receptor (benralizumab) were approved for patients exhibiting these characteristics, primarily those with T2-high inflammation. Low FEV_1_, high blood eosinophils and high exacerbations are the characteristics of patients who take advantage from a switch from omalizumab to anti IL-5/IL-5R therapy (3). Subsequently, the airway epithelium has been recognized not merely as a passive barrier, but as a key player in both inflammatory and immunological processes. Various epithelial triggers—such as allergens, viruses, bacteria, pollutants, and nanoparticles—can activate the epithelium, leading to the release of cytokines (e.g., IL-4 and IL-13) and alarmins (IL-25, IL-33, and thymic stromal lymphopoietin TSLP). In most cases, activation of group 2 innate lymphoid cells (ILC2s) contributes to epithelial responses, triggering downstream processes that frequently result in enhanced eosinophilic inflammation.

More recently, monoclonal antibodies targeting the IL-4 receptor alpha subunit—shared by both IL-4 and IL-13 (dupilumab)—and TSLP (tezepelumab) have been introduced for the treatment of patients with severe asthma (4, 5). As previously described, the majority of patients with severe asthma exhibit an eosinophilic inflammatory pattern, driven either by atopy with T2 cell activation or by the activation of ILC2s. In contrast, only a minority present a T2-low phenotype, characterized by neutrophilic or paucigranulocytic inflammation. In patients treated with tezepelumab and dupilumab, high blood eosinophils are associated with improvement of lung function, greater reduction of exacerbations, and higher possibility of oral corticosteroid (OCS) reduction (5, 6). Metanalysis of studies presented low- and high-grade evidence regarding predictors of response to biologics (7).

We previously investigated the differences among asthmatic patients with distinct inflammatory phenotypes and found that individuals with a “pure” eosinophilic pattern exhibited the most severe disease compared to those with neutrophilic or mixed (eosinophilic and neutrophilic) inflammation (8). Other studies have also demonstrated that a high neutrophil count is associated with a more severe asthma phenotype (9). However, no clear data are available on the impact of airway neutrophilic inflammation on the outcome of biologic therapy.

The aim of this study was to evaluate the impact of airway neutrophils on treatment response in patients with severe asthma receiving biologic therapy, in terms of exacerbation rate, lung function, and symptom control.

## 2 Materials and methods

### 2.1 Study design and setting

This retrospective observational study was conducted at an Italian referral asthma center (Istituti Clinici Scientifici Maugeri, IRCCS, Tradate, Italy) between 2018 and 2025, in accordance with the Declaration of Helsinki, and was approved by the Internal Review Board of ICS Maugeri, Tradate Institute (identifier: p3/16). This work was partially supported by the Ricerca Corrente Scheme of the Ministry of Health, Italy.

### 2.2 Patients and study procedures

Biologic-naïve patients with severe asthma, defined according to the Global Initiative for Asthma (GINA) recommendations (1), who initiated biologic therapy based on the Italian Medicines Agency (AIFA) eligibility criteria and had a minimum follow-up of 12 months, were considered for inclusion regardless of the specific biologic agent prescribed (omalizumab, mepolizumab, benralizumab, dupilumab, or tezepelumab).

Biological treatment was started in severe uncontrolled asthmatic patients who experienced at least two moderate/severe exacerbations in the previous year, according to Global Initiative for Asthma (GINA) recommendations and in accordance with local criteria established by the Italian Medicines Agency (AIFA). Omalizumab was prescribed for severe uncontrolled asthma patients, with FEV_1_ < 80% and allergic to one or more perennial aeroallergens (confirmed by skin prick testing or specific IgE), at the approved dosing range (once or twice per month) based on total serum IgE levels and body weight. Mepolizumab and benralizumab were prescribed for severe uncontrolled eosinophilic asthma, defined as a blood eosinophil count ≥300 cells/μL. Dupilumab was prescribed for severe uncontrolled asthma in presence of a type 2 inflammatory phenotype, defined by blood eosinophils ≥150/μL or fractional exhaled nitric oxide (FeNO) ≥25 ppb. Tezepelumab was prescribed regardless of allergic status, blood eosinophil count, or FeNO level, including in patients with non-eosinophilic and non-allergic asthma phenotypes.

Consecutive patients with a defined airway inflammatory pattern were enrolled. Assessment of airway inflammation was performed non-invasively through induced sputum analysis, routinely collected in clinically stable patients without asthma exacerbations in the month preceding the procedure. Sputum was induced with inhalation of 4.5% hypertonic saline, and samples were processed with dithiothreitol (DTT) in accordance with European Respiratory Society Recommendations (10, 11). Patients who provided an adequate induced sputum sample (enough material to obtain a cytospin with squamous cells <20%) (11) were included.

Patients were stratified into four groups according to their induced sputum inflammatory pattern: eosinophilic (eosinophils ≥3%), neutrophilic (neutrophils ≥61%), mixed granulocytic (eosinophil ≥3% and neutrophils ≥61%) and paucigranulocytic (eosinophils <3% and neutrophils <61%) (12).

Patients' electronic medical records were reviewed to assess clinical, functional, and inflammatory outcomes at baseline (T0) and at 6 (T6) and 12 months (T12) after initiation of the biologic therapy. The collected variables included baseline demographic characteristics (age, sex, body mass index, and smoking history); age at asthma onset (childhood-onset, <18 years; adult-onset, ≥18 years) (13); atopic status; symptom control; exacerbation frequency; ongoing pharmacological treatment; and comorbidities i.e., aspirin hypersensitivity, chronic rhinosinusitis with nasal polyps (CRSwNP), gastroesophageal reflux disease (GERD), arterial hypertension, bronchiectasis, emphysema, obstructive sleep apnea (OSA), and diabetes. Atopy was defined as sensitization to at least one common inhalant allergen, as determined by skin prick testing or serum-specific IgE measurement.

Asthma control was systematically assessed using validated questionnaires, including the Asthma Control Questionnaire (ACQ-6) (14) and the Asthma Control Test (ACT) (15, 16). Asthma exacerbations were documented and defined as episodes of symptom deterioration compared with the patient's stable condition, requiring treatment with oral corticosteroids for at least 3 days (1).

Lung function was evaluated by spirometry using MIR MiniSpir (MIR, Rome, Italy). Measured parameters included forced expiratory volume in one second (FEV_1_, expressed in liters and percentage of predicted), forced vital capacity (FVC, liters and % predicted), the FEV_1_/FVC ratio, and forced expiratory flow between 25% and 75% of FVC (FEF₂₅–₇₅_%_). The bronchodilator test was also conducted to determine the reversibility of airflow limitation after administration of an inhaler short-acting bronchodilator drug (salbutamol 400 µg) as a part of lung function tests. All tests were performed in accordance with American Thoracic Society/European Respiratory Society (ATS/ERS) recommendations (17), and results were normalized using Global Lung Initiative (GLI) reference equations (18).

Systemic inflammation was assessed with peripheral blood differential cell counts (UniCel DxH 800 hematology analyzer, Beckman Coulter, Pasadena, CA), while airway inflammation was evaluated through induced sputum analysis, as described above. Fractional exhaled nitric oxide (FeNO) was measured using the Vivatmo Pro analyzer (Bosch; COSMED, Rome, Italy) following ATS/ERS technical standards (19).

Therapeutic response was graded using a composite Biologic Asthma Response Score (BARS), integrating changes in annual exacerbation rate, daily oral corticosteroid dose, and ACT score. Patients were categorized as having an insufficient response, response, or good response, corresponding to scores of 0, 1, and 2, respectively (20).

### 2.3 Statistical analysis

Data are presented as mean ± standard deviation for normally distributed variables and as median with interquartile range for non-normally distributed variables. Normality of the data distribution was assessed using the Shapiro–Wilk test. At baseline (T0), comparisons among the three study groups were performed using analysis of variance (ANOVA) for normally distributed variables, or the Kruskal–Wallis test when assumptions were not met.

Within-group changes over time were analyzed using repeated-measures analysis of variance (ANOVA) for normally distributed data, while non-normally distributed data were analyzed using the Friedman test. *Post-hoc* pairwise comparisons were conducted with appropriate correction for multiple testing (Bonferroni correction).

Categorical variables were expressed as frequencies and percentages and compared between groups using the chi-square test, Fisher's exact test or McNemar test, as appropriate. All tests were two-tailed, and a *p*-value <0.05 was considered statistically significant. All analyses were performed using SPSS software (version 26, IBM Corp., Armonk, NY, USA).

## 3 Results

### 3.1 Baseline

A total of 143 patients with severe asthma underwent induced sputum collection before initiating biologic therapy. Of these, 30 patients failed to produce an adequate sputum sample for inflammatory cell analysis, resulting in a success rate of 79.0%. Therefore, we analyzed 113 patients with severe asthma who had undergone airway inflammatory phenotype characterization prior to the initiation of the biologic therapy. A paucigranulocytic pattern was identified in 10 patients, who were excluded from further analyses due to their small number. Among the remaining subjects (*n* = 103), 69.9% exhibited an eosinophilic pattern, 13.6% a mixed granulocytic pattern, and 16.5% a neutrophilic pattern. Patients were re-evaluated at 6 and 12 months following the initiation of biologic therapy. Baseline characteristics of the study population are summarized in Table 1.

**Table 1:** Patients’ characteristics at baseline before starting therapy with biologics.

Variables		Eosinophilic *n* = 72	Mixed granulocytic *n* = 14	Neutrophilic *n* = 17	*p* value	*Post hoc* Eos vs. Mixed	*Post hoc Eos vs.* Neutro	*Post hoc* Mixed vs. Neutro
Age (Years)		58 (48–64)	61 (52–68)	58 (53–72)	0.2534			
Male *n* (%)		33 (45.8)	6 (42.9)	4 (23.5)	0.2439			
BMI (Kg/m^2^)		23.7 (21.8–27.1)	27.1 (22.3–30.8)	25.0 (23.5–35.5)	0.1583			
Smoking habit	Never	43 (59.7)	11 (78.6)	8 (47.1)	0.2014			
	Ex-Smoker	29 (40.3)	3 (21.4)	9 (52.9)				
Pack Year		14.5 (5.00–30.00)	0.25 (0.00–11.25)	1.6 (0.00–10.5)	**0**.**0106**	0.0746	**0**.**0448**	0.6007
Late onset asthma		57 (79.2)	14 (100.0)	15 (88.2)	0.1337			
Disease duration, years		17,0 (10,0–34,7)	12,5 (9,7–24,2)	20,0 (3,5–29,0)	0.6978			
ACQ 6		1.00 (0.17–2.00)	0.50 (0.00–1.50)	1.50 (0.41–1.85)	0.2805			
ACT		21 (18–24)	20 (19–24)	18 (15–21)	0.0813			
Patients with exacerbation previous year *n* (%)		64 (90.1)	13 (86.7)	13 (76.5)	0.3122			
N° exacerbations previous year		2.0 (2.0–3.0)	2.0 (1.0–3.0)	2.0 (0.5–2.5)	0.0924			
Patients treated with OCS *n* (%)		16 (22.2)	6 (42.9)	7 (41.2)	0.2261			
Prednisone equivalent (mg)		6.2 (5.0–12.5)	5.0 (5.0–8.75)	7.5 (5–15)	0.5691			
Beclom. HFA equivalent (mcg)		600 (480–800)	480 (320–800)	688 (400–800)	0.2414			
Atopy *n* (%)		51 (70.8)	10 (70.4)	10 (58.8)	0.6146			
IgE tot KU/L		218.5 (119.5–571.2)	124.0 (69.5–924.7)	178.5 (56.4–382.2)	0.2551			
FeNO (ppb)		59 (33–113)	48 (25–155)	25.5 (12–61)	**0**.**0176**	1.0000	**0**.**0169**	0.1176
Blood leucocytes (10^9^/L)		7.09 (5.90–8.48)	7.86 (6.38–8.48)	7.44 (6.46–11.13)	0.4100			
Blood neutrophils (%)		53.3 (10.1)	54.5 (7.7)	61.1 (10.8)	**0**.**0153**	0.9124	**0**.**0118**	0.1453
Blood neutrophils (cells/µL)		3642,8 (2929,7–4743,8)	4,164.3 (3,457.3–4,677.5)	4,430.8 (3,405.5–8,057.3)	0.0912			
Blood eosinophils (%)		7.9 (5.0–9.6)	7.2 (5.25–10.35)	4.3 (1.75–5.7)	**0**.**0018**	1.0000	**0**.**0010**	**0**.**0401**
Blood eosinophils (cells/µL)		596.6 (323,5–737,6)	536.9 (346.4–743.4)	281.1 (113.0–473.8)	**0**.**0122**	1.0000	**0**.**0075**	0.0695
Blood lymphocytes (%)		29.5 (8.2)	28.4 (7.4)	25.4 (9.5)	0.1973			
Blood lymphocyte (cells/µL)		1,923.7 (1,707.2–2,483.4)	2,140.0 (1,662.2–2,591.6)	1,958.4 (1,350.7–2,338.1)	0.5470			
Neutrophils/Lymphocytes (NLR)		1.82 (1.35–2.18)	1.86 (1.41–2.76)	2.44 (1.67–3.22)	**0**.**0490**	1.0000	**0**.**0452**	0.0701
IS cells (10^4^/mL)		103.0 (49.5–210.5)	340.0 (193.2–660.0)	317.5 (128.7–566.5)	**<0**.**0001**	**0**.**0061**	**0**.**0055**	1.0000
IS viability (%)		74.0 (52.7–83.0)	89.0 (82.1–91.0)	94.0 (83.3–97.2)	**<0**.**0001**	**0**.**0382**	**<0**.**0001**	1.0000
IS macrophages (%)		9.2 (5.1–16.9)	3.6 (1.6–10.2)	7.0 (1.8–12.9)	**0**.**0258**	**0**.**0427**	0.3632	1.0000
IS neutrophils (%)		19.7 (7.2–42.4)	80.4 (71.8–90.0)	87.6 (83.0–95.1)	**<0**.**0001**	**<0**.**0001**	**<0**.**0001**	1.0000
IS eosinophils (%)		58.7 (32.4–78.0)	10.8 (6.3–15.4)	0.6 (0.0–2.0)	**<0**.**0001**	**<0**.**0001**	**<0**.**0001**	0.1437
IS eosinophils (10^4^)/mL		50.0 (18.8–107.0)	38.6 (18.7–66.5)	3.1 (0.0–7.7)	**<0**.**0001**	**<0**.**0001**	**<0**.**0001**	1.0000
IS lymphocytes (%)		0.7 (0.2–1.8)	0.3 (0.1–0.5)	1.0 (0.0–2.0)	0.2862			
IS epithelial cells (%)		5.7 (2.7–11.3)	1.4 (0.5–2.5)	1.8 (0.3–4.1)	**<0**.**001**	**<0**.**0001**	**<0**.**0001**	1.0000
Pre FEV_1_ (L)		2.36 (0.76)	2.39 (0.91)	1.78 (0.89)	0.0534			
Pre FEV_1_ (%)		77.0 (18.3)	80.7 (19.8)	69.4 (28.6)	0.3000			
Pre FVC (L)		3,27 (2,67–3,83)	3.17 (2.59–3.73)	2.69 (1.81–3.21)	**0**.**0293**	1.0000	**0**.**0254**	1.0000
Pre FVC (%)		88.6 (15.1)	88.9 (17.0)	80.8 (21.6)	0.2497			
Pre FEV_1_/FVC		67.9 (10.3)	70.5 (9.4)	64.2 (13.6)	0.2196			
Pre FEF 25–75 (L/s)		1.38 (1.04–2.08)	1.97 (0.96–2.84)	1.13 (0.73–2.89)	0.4670			
Pre FEF 25–75 (%)		56.5 (36.8–77.2)	73.9 (42.8–107.4)	47.6 (31.4–96.9)	0.2786			
Post FEV_1_ (L)		2.48 (1.79–2.95)	2.44 (1.76–2.82)	2.13 (1.43–2.70)	0,2066			
Post FEV_1_ (%)		81.5 (69.7–96.1)	85.1 (65.3–105.8)	81.6 (60.4–98.6)	0.7722			
Post FVC (L)		3.32 (2.69–4.03)	3.12 (2.73–3.66)	2.84 (2.30–3.37)	0.1620			
Post FVC (%)		91.5 (79.4–101.4)	89.8 (77.5–103.4)	89.1 (81.9–97.5)	0.9389			
Post FEV_1_/FVC (%)		72.4 (63.8–79.3)	78.2 (65.8–82.7)	68.0 (60.2–83.7)	0.5183			
Post FEF 25–75 (L/s)		1.86 (1.28–2.40)	1.64 (1.32–3.76)	1.65 (0.85–3.53)	0.5361			
Post FEF 25–75 (%)		63.6 (42.1–91.3)	77.1 (52.4–120.2)	59.0 (40.9–125.5)	0.4099			
ASA Hypersensitivity *n* (%)		15 (20.8)	3 (21.4)	2 (11.7)	0.7392			
CRwNP *n* (%)		56 (77.8)	9 (64.3)	4 (23.53)	**<0**.**0001**	0.9501	**<0**.**0001**	**0**.**0158**
GERD *n* (%)		29 (40.3)	2 (14.3)	8 (47.1)	0.1289			
Arterial Hypertension *n* (%)		33 (46.5)	6 (40.0)	9 (52.9)	0.8308			
Bronchiectasis *n* (%)		24 (33.3)	5 (35.7)	7 (41.2)	0.8285			
Emphysema *n* (%)		8 (11.1)	1 (7.1)	4 (23.5)	0.2964			
OSA *n* (%)		13 (18.1)	1 (7.1)	7 (41.2)	**0**.**0432**	1.0000	0.0672	**0**.**0406**
Diabetes *n* (%)		2 (2.8)	0 (0.0)	0 (0.0)	0.4981			
Mepolizumab *n* (%)		29 (40.3)	2 (14.3)	7 (41.2)	0.001			
Omalizumab *n* (%)		11 (15.3)	2 (14.3)	2 (11.8)				
Benralizumab *n* (%)		21 (29.2)	7 (50.0)	2 (11.8)				
Dupilumab *n* (%)		10 (13.9)	3 (21.4)	1 (5.8)				
Tezepelumab *n* (%)		1 (1.3)	0 (0.0)	5 (29.4)				

Eos, eosinophilic patter; Mixed, mixed granulocytic pattern; Neutro, neutrophilic pattern; BMI, body mass index; ACQ, asthma control questionnaire; ACT, asthma control test; Beclom., beclometasone; OCS, oral corticosteroid; HFA, hydrofluoroalkane; FeNO, fractional exhaled nitric oxide; IS, induced sputum¸ Pre, pre-bronchodilator measurements; FEV_1_, forced expiratory volume in 1 second; FVC, forced vital capacity, FEF, forced expiratory flow; ASA, aspirin sensitive asthma; CRwNP, chronic rhinosinusitis with nasal polyps; GERD, gastroesophageal reflux disease; OSA, obstructive sleep apnea.

The distribution of peripheral blood leukocytes differed according to the airway inflammatory phenotype. Blood eosinophil counts and percentages were higher in the eosinophilic group than in the neutrophilic group, whereas patients with a mixed granulocytic pattern showed increased eosinophil percentages only, compared with neutrophilic patients. Neutrophilic subjects exhibited higher blood neutrophil percentages and an elevated neutrophil-to-lymphocyte ratio (NLR) compared with eosinophilic subjects. FeNO levels—but not serum total IgE—were higher in eosinophilic and mixed granulocytic patients than in those with a neutrophilic pattern. Total induced sputum cell counts were higher in both mixed granulocytic and neutrophilic phenotypes than in eosinophilic patients, as was sputum cell viability. The proportion of induced sputum macrophages was greater in eosinophilic than in mixed granulocytic subjects. Increased epithelial cell counts were observed in eosinophilic and mixed granulocytic patients compared with neutrophilic patients.

With regard to lung function, FVC (L) was lower in neutrophilic than in eosinophilic patients, whereas FEV_1_ tended to be lower as well, although the difference did not reach statistical significance. Chronic rhinosinusitis with nasal polyposis was more frequent in eosinophilic and mixed granulocytic patients, whereas the prevalence of obstructive sleep apnea (OSA) was higher in the neutrophilic group compared with mixed granulocytic patients.

In the neutrophilic group, most patients (14/17, 82.3%) exhibited T2-high biomarkers based on the prescription criteria in our country, despite the absence of increased sputum eosinophils: 10 were atopic, 8 had elevated FeNO levels (≥25 ppb), and 12 had blood eosinophil counts ≥300 cells/µL in the absence of OCS therapy or ≥150 cells/µL while receiving OCS therapy.

Thirty-eight patients were treated with mepolizumab, 30 with benralizumab, 15 with omalizumab, 14 with dupilumab, and 6 with tezepelumab, according to their baseline clinical and inflammatory characteristics as described in the Methods section. Distribution of biologics in the different groups is shown in Table 1.

### 3.2 Follow-up

Patients were re-evaluated at 6 and 12 months after the initiation of biologic therapy. A significant reduction in sputum production was observed in eosinophilic and neutrophilic subjects (*p* < 0.001 and *p* = 0.015, respectively), whereas a trend toward reduced sputum production was noted in mixed granulocytic subjects (*p* = 0.072). Data stratified according to baseline airway inflammatory phenotype are presented in Tables 2–4.

**Table 2:** Clinical and inflammatory variables of patients with eosinophilic pattern before and after 6 and 12 months of biologic therapy. Data are referred to patients with all the variable at the three time points.

Eosinophilic patients *n* = 72	T0	T6	T12	p	*p* T0 vs. T6	*p* T0 vs. T12	*p* T6 vs. T12
ACQ 6 *n* = 68	1.00 (0.17–2.00)	0.00 (0.00–0.50)	0.09 (0.00–0.33)	**<0**.**0001**	**<0**.**0001**	**<0**.**0001**	0,9738
ACT *n* = 68	22 (18–24)	24 (24–25)	25 (24–25)	**<0**.**0001**	**<0**.**0001**	**<0**.**0001**	0,2181
Exacerb. rate since biologics start *n* = 72	2.0 (2.00–3.00)	0.00 (0.00–0.00)	0.00 (0.00–0.00)	**<0**.**0001**	**<0**.**0001**	**<0**.**0001**	1.0000
Patients treated with OCS *n* (%)	16 (22.2)	16 (22.2)	10 (7.2)	0.097			
Prednisone equivalent (mg) *n* = 72	10.0 (6.2–12.5)	3.0 (2.5–5.0)	1.2 (0.0–5.0)	**0**.**005**	**0**.**033**	**0**.**002**	0.3500
Beclom. HFA equivalent (mcg) *n* = 72	600 (480–800)	600 (480–800)	600 (400–800)	0,5766			
FeNO (ppb) *n* = 38	67 (34–111)	42 (23–53)	38 (24–63)	**0**.**0058**	**<0**.**0001**	**0**.**0112**	0,3840
Blood leukocytes (10^9^/L) *n* = 61	7.10 (5.98–8.42)	6.59 (5.61–8.05)	6.58 (5.62–7.57)	**0**.**0069**	**0**.**0146**	**0**.**0002**	0,2754
Blood neutrophils (%) *n* = 61	53.3 ± 10.2	57.1 ± 9.6	58.3 ± 7.8	0.7600			
Blood neutrophils (cells/µL) *n* = 61	3,632.8 (2,882.4–4,757.5)	3,726.6 (2,899.8–4,690.2)	3,812.5 (3,046.4–4,856.2)	0,5515			
Blood eosinophils (%) *n* = 61	7.9 ± 4.3	3.2 ± 4.8	2.2 ± 3.3	**<0**.**0001**	**<0**.**0001**	**<0**.**0001**	1.0000
Blood eosinophils (cells/µL) *n* = 61	491.5 (326.3–729.5)	61.6 (29.1–271.6)	50.6 (0.0–172.8)	**<0**.**0001**	**<0**.**0001**	**<0**.**0001**	**0.0019**
Blood lymphocytes (%) *n* = 61	29.4 (25.2–35.1)	29.4 (25.6–35.0)	29.1 (24.9–33.6)	0.9959			
Blood lymphocytes (cells/µL) *n* = 61	1,922.1 (1,714.7–2,419.9)	1,914.7 (1,587.0–2,208.2)	1,870.0 (1,598.4–2,189.5)	0.0823			
Neutrophils/Lymphocytes NRL *n* = 61	1.87 (1.41–2.37)	1.93 (1.64–2.51)	2.01 (1.58–2.53)	0.5080			
IS cells (10^4^/mL) *n* = 16	98.5 (61.2–204.2)	124.5 (68.2–282.7)	97.0 (51.5–208.0)	0.5698			
IS viability (%) *n* = 16	59.0 (46.4–77.2)	82.7 (58.2–89.0)	75.0 (56.0–90.0)	0.3973			
IS macrophages (%) *n* = 23	8.5 (5.3–15.8)	21.6 (11.3–50.7)	18.1 (9.1–32.8)	**0**.**0405**	**0,0014**	0,1288	0,6143
IS neutrophils (%) *n* = 23	17.9 (9.8–29.3)	31.8 (26.7–71.2)	61.8 (35.9–75.9)	**0**.**0281**	**<0**.**0001**	**<0**.**0001**	0,3065
IS eosinophils (%) *n* = 23	60.0 (31.8–78.3)	1.4 (0.0–6.0)	0.9 (0.1–8.8)	**<0**.**0001**	**<0**.**0001**	**0,0001**	0,8446
IS eosinophils (10^4^/mL) *n* = 23	50.0 (26.2–104.7)	0.91 (0.0–8.5)	0.9 (0.1–10.6)	**0**.**0006**	**<0**.**0001**	**0,0001**	0,8647
IS lymphocytes (%) *n* = 23	0.6 (0.2–1.8)	0.8 (0.3–1.7)	0.5 (0.2–1.1)	0.1337			
IS epithelial cells (%) *n* = 23	6.4 (2.8–11.3)	5.1 (2.10–7.3)	5.5 (2.3–8.8)	0.9886			
Pre FEV_1_ (L) *n* = 55	2.29 ± 0.74	2.62 ± 0.81	2.68 ± 0.88	0.8074			
Pre-FEV_1_ (%) *n* = 55	82,0 (69.0–94.0)	87.7 (74.0–102.7)	90.2 (77.5–103.1)	**0**.**0030**	**0**.**0090**	**0**.**0010**	0.5600
Pre FVC (L) *n* = 55	3.35 ± 1.02	3.62 ± 1.01	3.64 ± 1.10	0.9958			
Pre FVC (%) *n* = 55	89.1 (77.7–101.3)	95.4 (83.1–104.1)	95.0 (86.0–104.5)	0.0610			
Pre FEV_1_/FVC *n* = 55	68.0 (61.7–75.0)	72.2 (66.0–77.4)	73.3 (66.9–79.7)	**0**.**0002**	**<0**.**0001**	**<0**.**0001**	0,6853
Pre FEF 25–75 (L/s) *n* = 55	1.38 (1.04–2.03)	2.04 (1.20–2.78)	2.04 (1.33–2.64)	0.8863			
Pre FEF 25–75 (%) *n* = 55	56.6 (43.5–85.8)	69.4 (52.1–103.3)	74.4 (55.9–97.5)	**0**.**0020**	**0**.**0010**	**0**.**0010**	0.7370

ACQ, asthma control questionnaire; ACT, asthma control test; Beclom., beclometasone; OCS, oral corticosteroid; HFA, hydrofluoroalkane; FeNO, fractional exhaled nitric oxide; IS, induced sputum¸ Pre, pre-bronchodilator measurements; FEV_1_, forced expiratory volume in 1 second; FVC, forced vital capacity, FEF, forced expiratory flow.

**Table 3:** Clinical and inflammatory variables of patients with mixed-granulocytic pattern before and after 6 and 12 months of biologic therapy. Data are referred to patients with all the variable at the three time points.

Mixed granulocytic patients *n* = 14	T0	T6	T12	p	*p* T0 vs. T6	*p* T0 vs. T12	*p* T6 vs. T12
ACQ 6 *n* = 13	0.42 (0.00–1.42)	0.00 (0.00–0.00)	0.00 (0.00–0.17)	**0**.**0009**	**0**.**0076**	**0**.**0178**	0.0679
ACT *n* = 13	21 (19–24)	25 (25–25)	25 (24–25)	**0**.**0002**	**0**.**0032**	**0**.**0074**	0.0707
Exacerb. rate since biologics start *n* = 14	2.00 (2.00–2.50)	0.00 (0.00–0.00)	0.00 (0.00–0.00)	**<0**.**0001**	**0**.**005**	**0**.**001**	1.000
Patients treated with OCS *n* (%)	6 (42.9)	6 (42.9)	3 (21.4)	0.2470			
Prednisone equivalent (mg) *n* = 14	12.50 (3.75–15.0)	5.0 (3.75–7.25)	5.0 (3.75–7.25)	0.3680			
Beclom. HFA equivalent (mcg) *n* = 14	480 (320–800)	480 (320–800)	800 (400–800)	0.0970			
FeNO (ppb) *n* = 7	48 (34–137)	47 (37–84)	49 (32–80)	0.7720			
Blood leukocytes (10^9^/L) *n* = 13	7.64 ± 1.74	6.80 ± 1.54	6.89 ± 1.29	**0**.**0234**	**0**.**0444**	**0**.**0337**	0.4542
Blood neutrophils (%) *n* = 13	55.0 ± 8.1	57.4 ± 9.5	58.1 ± 7.4	0.3750			
Blood neutrophils (cells/µL) *n* = 13	4,164.3 (3,457.6–4,677.0)	3,744.8 (2,781.3–4,259.4)	3,673.6 (3,438.3–3,975.3)	0.3385			
Blood eosinophils (%) *n* = 13	8.2 ± 3.9	2.8 ± 5.5	2.2 ± 3.9	**0**.**0024**	**0**.**0106**	**0**.**0051**	0.6858
Blood eosinophils (cells/µL) *n* = 13	622.6 ± 327.1	196.0 ± 389.0	150.6 ± 283.9	**0**.**0024**	**0**.**0147**	**0**.**0074**	0.8658
Blood lymphocytes (%) *n* = 13	27.22 ± 7.31	28.89 ± 8.56	28.62 ± 6.66	0.6002			
Blood lymphocytes (cells/µL) *n* = 13	2,054.9 ± 588.2	1,926.3 ± 603.9	1982.8 ± 600.5	0.5596			
Neutrophils/Lymphocytes NRL *n* = 13	1.97 (1.43–2.91)	1.99 (1.31–3.20)	2.10 (1.53–2.42)	0.9200			
IS cells (10^4^/mL) *n* = 3	660.0 (340.0–775.0)	422.0 (152.0–650.0)	530.0 (405.0–540.0)	0.7165			
IS viability (%) *n* = 3	90.0 (89.0–91.0)	91.0 (85.0–93.0)	92.5 (88.9–95.0)	1.0000			
IS macrophages (%) *n* = 3	5.6 (3.3–10.3)	17.1 (8.9–28.9)	4.6 (1.9–34.0)	0.2636			
IS neutrophils (%) *n* = 3	80.4 (67.7–84.5)	76.4 (67.6–86.0)	90.7 (58.2–94.0)	0.7165			
IS eosinophils (%) *n* = 3	12.4 (9.4–14.6)	0.0 (0.0–0.2)	0.0 (0.0–1.0)	0.0859			
IS eosinophils (10^4^/mL) *n* = 3	35.4 (3.1–50.0)	0.0 (0.0–0.0)	5.1 (0.0–7.7)	0.1561			
IS lymphocytes (%) *n* = 3	0.3 (0.2–0.5)	0.7 (0.5–1.4)	1.1 (0.1–1.3)	0.9131			
IS epithelial cells (%) *n* = 3	1.5 (1.1–2.2)	4.2 (1.5–7.8)	4.7 (1.5–6.0)	0.0970			
Pre FEV_1_ (L) *n* = 10	2.43 ± 0.94	2.61 ± 0.87	2.61 ± 0.89	**0**.**0385**	**0**.**0184**	**0**.**0225**	0.8457
Pre FEV_1_ (%) *n* = 10	84.0 (69.3–101.8)	92.4 (81.1–103.2)	92.4 (80.7–106.3)	**0**.**0006**	**0**.**025**	**0**.**002**	0.371
Pre FVC (L) *n* = 10	3.35 ± 1.20	3.49 ± 1.13	3.52 ± 1.05	0.0673			
Pre FVC (%) *n* = 10	95.2 (85.0–103.8)	98.4 (93.5–101.6)	98.7 (93.5–102.8)	0.1460			
Pre FEV_1_/FVC *n* = 10	72.1 ± 7.7	75.3 ± 8.6	73.5 ± 9.3	0.1420			
Pre FEF 25–75 (L/s) *n* = 10	2.16 ± 0.90	2.51 ± 1.04	2.29 ± 1.05	0.3008			
Pre FEF 25–75 (%) *n* = 10	95.2 (53.9–120.2)	93.6 (62.5–108.1)	107.0 (77.6–121.4)	0.2360			

ACQ, asthma control questionnaire; ACT, asthma control test; Beclom., beclometasone; OCS, oral corticosteroid; HFA, hydrofluoroalkane; FeNO, fractional exhaled nitric oxide; IS, induced sputum¸ Pre, pre-bronchodilator measurements; FEV_1_, forced expiratory volume in 1 second; FVC, forced vital capacity, FEF, forced expiratory flow.

**Table 4:** Clinical and inflammatory variables of patients with neutrophilic pattern before and after 6 and 12 months of biologic therapy. Data are referred to patients with all the variable at the three time points.

Neutrophilic patients *n* = 17	T0	T6	T12	*p*	*p* T0 vs. T6	*p* T0 vs. T12	*p* T6 vs. T12
ACQ 6 *n* = 16	1.35 ± 0.95	0.57 ± 0.81	0.76 ± 0.91	**0**.**0102**	**0**.**0015**	0.0509	0.4090
ACT *n* = 16	18 ± 4.0	22 ± 4	22 ± 4	**0**.**0180**	**0,0169**	**0**.**0048**	0.8781
Exacerb. rate since biologics start *n* = 17	2.0 (1.25–2.00)	0.00 (0.00–1.00)	0.00 (0.00–1.00)	**<0**.**0001**	**0**.**004**	**0**.**004**	1.000
Patients treated with OCS *n* (%) *n* = 17	7 (41.2)	6 (35.3)	5 (29.4)	0.8670			
Prednisone equivalent (mg) *n* = 17	15.00 (7.50–15.00)	5.00 (2.5–12.50)	5.00 (2.5–12.50)	0.0500			
Beclom. HFA equivalent (mcg) *n* = 17	800 (400–800)	800 (600–800)	800 (600–800)	0.2683			
FeNO (ppb) *n* = 9	21 (11–98)	21 (13–98)	25 (20–51)	0.9131			
Blood Leukocytes (10^9^/L) *n* = 16	8.58 ± 2.87	7.33 ± 2.14	6.54 ± 1.16	**0**.**0104**	0.0627	**0**.**0072**	0.1044
Blood neutrophils (%) *n* = 16	61.1 ± 10.8	60.6 ± 9.4	57.3 ± 8.0	0.8948			
Blood neutrophils (cells/µL) *n* = 16	4,430.8 (3,483.1–7,819.6)	3,976.6 (3,272.9–4,282.04	4,019.0 (3,097.1–4,477.1)	0.1561			
Blood eosinophils (%) *n* = 16	4.2 ± 2.7	2.1 ± 2.8	1.9 ± 2.8	**0**.**0090**	0.0661	0.0656	0.6247
Blood eosinophils (cells/µL) *n* = 16	325.6 ± 210.5	141.2 ± 189.1	116.4 ± 161.1	**0**.**0063**	**0**.**0252**	**0**.**0132**	0.8613
Blood lymphocytes (%) *n* = 16	25.4 ± 9.5	28.0 ± 8.0	31.4 ± 7.1	**0**.**0424**	0.3290	**0**.**0166**	0.2455
Blood lymphocytes (cells/µL) *n* = 16	1,962.6 (1,500.3–2,265.1)	1,934.2 (1,666.4–2,231.2)	1,986.6 (1,603.3–2,517.6)	0.8135			
Neutrophils/Lymphocytes NRL *n* = 16	2.36 (1.59–3.25)	1.92 (1.55–2.98)	1.75 (1.52–2.28)	0.5050			
IS cells (10^4^/mL) *n* = 7	349.5 (211.2–432.2)	549.0 (225.6–695.0)	241.0 (143.5–351.5)	0.6271			
IS viability (%) *n* = 7	96.0 (85.0–97.0)	94.0 (90.2–96.2)	87.5 (73.2–93.0)	0.4204			
IS macrophages (%) *n* = 9	7.6 (1.9–10.6)	8.4 (6.2–10.9)	5.8 (2.3–16.1)	0.4677			
IS neutrophils (%) *n* = 9	87.1 (84.8–94.2)	83.1 (54.7–90.5)	79.4 (51.7–88.8)	0.0872			
IS eosinophils (%) *n* = 9	1.9 (0.4–2.0)	0.0 (0.0–0.6)	1.2 (0.5–3.2)	0.4378			
IS eosinophils (10^4^/mL) *n* = 7	5.4 (2.9–7.3)	0.7 (0.0–7.1)	2.1 (1.0–5.5)	0.6065			
IS lymphocytes (%) *n* = 9	0.9 (0.0–2.0)	0.9 (0.1–1.3)	1.1 (0.5–1.3)	0.8338			
IS epithelial cells (%) *n* = 9	1.7 (0.3–4.1)	2.3 (1.7–4.9)	3.5 (1.2–5.0)	0.1947			
Pre FEV_1_ (L) *n* = 13	1.70 ± 0.95	1.76 ± 0.90	1.77 ± 0.95	0.8002			
Pre FEV_1_ (%) *n* = 13	62.1 (38.7–99.0)	78.6 (46.0–96.0)	74.5 (32.2–97.1)	0.4110			
Pre FVC (L) *n* = 13	2.54 ± 1.01	2.66 ± 1.01	2.65 ± 1.10	0.3051			
Pre FVC (%) *n* = 13	91.0 (60.0–94.9)	98.0 (67.0–100.0)	91.0 (57.0–98.6)	0.1090			
Pre FEV_1_/FVC *n* = 13	62.5 ± 13.6	63.4 ± 12.9	63.0 ± 13.7	0.8917			
Pre FEF 25–75 (L/s) *n* = 13	1.53 ± 1.34	1.49 ± 1.19	1.66 ± 1.25	0.4378			
Pre FEF 25–75 (%) *n* = 13	50.3 (27.5–121.4)	55.9 (38.1–107.5)	49.9 (32.0–112.5)	0.8460			

ACQ, asthma control questionnaire; ACT, asthma control test; Beclom., beclometasone; OCS, oral corticosteroid; HFA, hydrofluoroalkane; FeNO, fractional exhaled nitric oxide; IS, induced sputum¸ Pre, pre-bronchodilator measurements; FEV_1_, forced expiratory volume in 1 second; FVC, forced vital capacity, FEF, forced expiratory flow.

In eosinophilic subjects, both ACQ-6 and ACT scores improved at 6 and 12 months compared with baseline. Blood leukocyte counts, blood eosinophil counts, FeNO levels, and the percentages of eosinophils and lymphocytes in induced sputum significantly decreased, whereas the FEV_1_/FVC ratio increased after 6 months. Small airway function evaluated as FEF 25–75 (%) improved after biologics.

All these improvements remained significant at 12 months; only blood eosinophil counts showed a further significant reduction between 6 and 12 months. The percentages of macrophages and neutrophils in induced sputum varied as a consequence of the eosinophil decrease. Exacerbation rates were significantly reduced at both 6 and 12 months of biologic therapy compared with baseline, and the OCS dose was also markedly reduced (87.5% reduction).

In subjects with a mixed granulocytic pattern, both ACQ-6 and ACT scores improved at 6 and 12 months compared with baseline. Blood leukocyte counts, as well as blood eosinophil percentages and counts, decreased, while FEV_1_ (%) and FEV_1_ (L) increased at both T6 and T12. Induced sputum eosinophils showed a decreasing trend at T6 and T12, although the changes were not statistically significant, as only a few subjects in this group continued to produce sputum after initiation of biologic therapy. Exacerbation rates significantly decreased at both time points following the initiation of biologic therapy, accompanied by a reduction in OCS dose (60% reduction). None of the variables showed further improvement between T6 and T12.

In subjects with a neutrophilic pattern, ACQ-6 scores significantly decreased only at T6 compared with baseline, while ACT scores increased at both T6 and T12. Blood leukocyte counts decreased only at T12, blood eosinophil counts decreased at both T6 and T12, and blood lymphocyte percentages decreased only after 12 months. Exacerbation rates were significantly reduced at both 6 and 12 months following the initiation of biologic therapy; however, although the OCS dose was reduced (66.7% reduction), the change did not reach statistical significance. None of the pulmonary function parameters improved at either T6 or T12, and no variables showed further significant changes between these time points.

**Figure 1:**
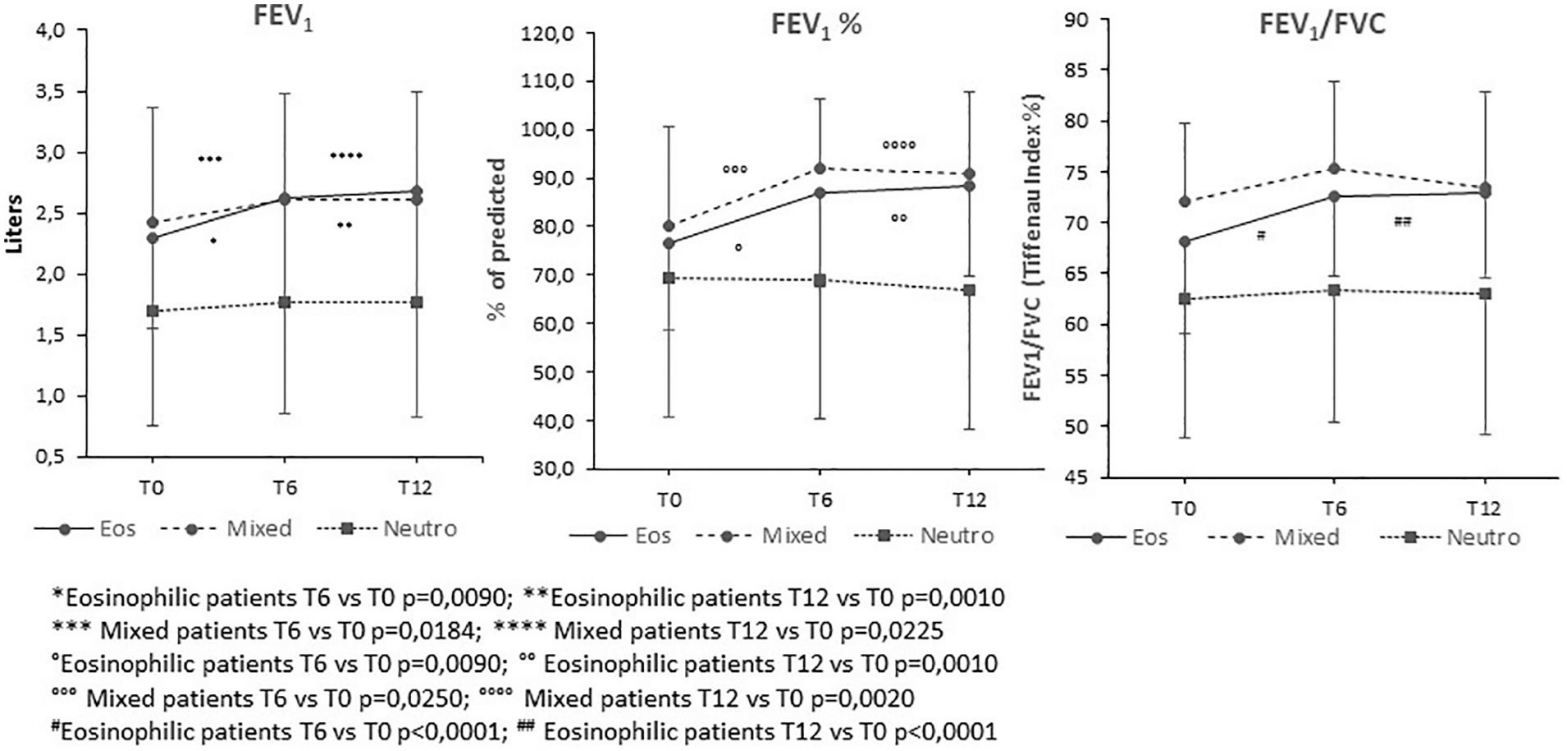
Follow-up of lung function parameters in severe asthmatic patients treated with biologics. FEV_1_ (L), FEV_1_ (% pred) and FEV_1_/FVC in patients with severe asthma at baseline (T0) and after 6 and 12 months of biologic treatment, stratified according to baseline sputum inflammatory pattern. Data are presented as mean and standard deviation. Eos, eosinophilic patter; Mixed, mixed granulocytic pattern; Neutro, neutrophilic pattern; FEV_1_, forced expiratory; FEV_1_/FVC ratio, forced expiratory volume in 1 second/forced vital capacity.

Lung functions FEV_1_/FVC and FEV_1_ (L) at T0 and during the follow-up are shown in the Figure 1 and as single data plots in Supplementary Figure S1.

Neutrophilic patients exhibited significantly different ΔFEV_1_ (T12–T0) and ΔACQ-6 (T12–T0) values compared with eosinophilic patients. The changes (Δ) for all variables considered are reported in Supplementary Table S1.

When comparing therapeutic response, assessed using the BARS score (20, 21), a significant difference was observed among the three groups: eosinophilic (mean 1.90 ± 0.22), mixed granulocytic (mean 1.9 ± 0.09), and neutrophilic (mean 1.59 ± 0.59) (*p* = 0.006, ANOVA). *Post hoc* analysis revealed significant differences between eosinophilic and neutrophilic patients (*p* = 0.013) and between mixed granulocytic and neutrophilic patients (*p* = 0.007).

## 4 Discussion

In this retrospective study, we evaluated symptoms, clinical and inflammatory parameters in patients with severe asthma treated with biologic therapy for 12 months, divided according to their airway inflammatory phenotype prior to therapy initiation. Patients were in a stable condition for at least one month, as indicated by ACQ-6 values, before undergoing induced sputum collection. All enrolled patients received their first biologic therapy. Biologic therapies have demonstrated sustained effectiveness over the past decade, with meaningful improvements in asthma-related outcomes. However, the impact of central airway inflammatory patterns on treatment response remains poorly explored, largely because current biologic prescribing and predictive criteria do not incorporate information on airway inflammatory phenotypes in the selection of biologic therapy.

At baseline, 69.9% of the enrolled patients exhibited an eosinophilic pattern, 13.6% a mixed granulocytic pattern, and 16.5% a neutrophilic pattern. Paucigranulocytic subjects were too few and were therefore excluded from the analysis.

Patients with a neutrophilic inflammatory pattern exhibited lower lung function than eosinophilic patients, particularly in terms of FVC (L), and showed a trend toward lower FEV_1_ (L), although differences in FEV_1_% predicted and FVC% predicted did not reach statistical significance. With regard to comorbidities, the neutrophilic group showed a higher prevalence of OSA and a lower prevalence CRSwNP compared with the eosinophilic and mixed granulocytic groups, in line with previous studies (8). Interestingly, 14/17 (82.7%) patients with neutrophilic airway inflammation, had high T2 biomarkers as high blood eosinophils and/or high IgE and/or high FeNO. However, FeNO and blood eosinophils were lower in the neutrophilic group compared with the eosinophilic group.

We previously compared the characteristics of asthmatic patients of varying severity according to their airway inflammatory phenotypes (8). We found that the presence of neutrophils was more frequent in older or obese patients. In the present study, we focused exclusively on severe asthmatics with different inflammatory phenotypes. The three inflammatory groups did not differ in terms of age or BMI. In contrast, in neutrophilic severe asthmatic patients, we confirmed increased total sputum cell counts and cell viability, which are typically markers of rapid cell recruitment and may indicate potential colonization or infection of the bronchial airways (22).

All three groups experienced improved outcomes after 6 months of treatment, which were generally maintained at 12 months, with further improvement between 6 and 12 months observed only in blood eosinophil counts in the eosinophilic group. All patients experienced a significant reduction in sputum production after 12 months of biologic therapy, suggesting an effect on mucus production independent of the underlying inflammatory phenotype. Patients in the eosinophilic group showed improvement in both inflammatory and functional parameters at T6, which was maintained at T12, along with improvements in symptom scores, OCS reduction, and exacerbation rates. Patients with a mixed granulocytic phenotype also showed improvements at T6 and T12, similar to eosinophilic patients, but the reduction in OCS dose was not statistically significant. Patients with a neutrophilic phenotype showed improvements in symptoms, exacerbation rates, and blood eosinophil counts, confirming the relatively high baseline blood eosinophil levels observed in a subset of these patients. However, no significant changes were detected in lung function, and the reduction in OCS use did not reach statistical significance. FeNO levels decreased only in eosinophilic patients, remaining elevated in both mixed granulocytic and neutrophilic subjects.

Therapeutic options for neutrophilic asthma are still lacking, although anti-TSLP therapy has shown efficacy in both T2-high and T2-low asthma (4). Neutrophilic airway inflammation can only be non-invasively assessed through sputum analysis and may encompass patients with heterogeneous inflammatory mechanisms. In our cohort, most neutrophilic patients exhibited T2 biomarkers: 10 were atopic, 8 had elevated FeNO levels, and 12 had blood eosinophil counts >300 cells/µL in the absence of OCS therapy or >150 cells/µL while receiving OCS therapy. These neutrophilic patients experienced reductions in blood eosinophils following biologic therapy, but did not show statistically significant improvements in lung function or significant reductions in sputum neutrophilic inflammation. Most of these patients phenotyped using blood eosinophils, IgE, or FeNO, are classified as T2-high. However, the presence of neutrophilic bronchial inflammation shows compartmentalization, with discrepancies between systemic and airway inflammation. The neutrophilic pattern remained stable over time, as patients exhibited persistent neutrophilic airway inflammation at 6 and 12 months, with a non-statistically significant trend toward reduction. No significant improvements in lung function or reductions in OCS dose were observed following treatment. This suggests that evaluation of airway inflammation after biologic therapy can reveal persistent neutrophilic inflammation, which may underlie the lack of improvement in certain clinical outcomes. Further studies are needed to confirm these suggestions.

Furthermore, when therapeutic resolution was assessed using the BARS score (20, 21), neutrophilic patients had significantly lower scores compared with eosinophilic and mixed granulocytic patients.

A recent study confirmed the coexistence of T2 and T1 inflammation in severe asthmatic patients treated with biologics. The authors demonstrated that a subset of T2-high patients continued to experience exacerbations despite anti–IL-5 therapy. These patients had longer disease duration, poorer lung function, and increased sputum protein levels, including elevated markers of neutrophil activity (23). This high protein expression was already present at baseline and persisted throughout follow-up, without being altered by biologic therapy. Our data support these findings, showing that in patients with sustained airway neutrophilia, the efficacy of biologic treatment, not yet targeted to neutrophilic inflammation, is lower than in patients with purely eosinophilic inflammation, particularly with respect to lung function recovery.

The neutrophilic subset of asthmatic patients is more complex than can be represented by sputum neutrophil counts alone, as demonstrated by a study from the U-BIOPRED group. At least three distinct clusters have been identified within neutrophilic patients. These clusters are characterized by increased sputum expression of IL-2, IFN-α, and IFN-γ, along with decreased expression of IL-13, IL-4, retinoic acid receptor *α*, and other biomarkers, reflecting the activation and downregulation of distinct immunologic pathways (24).

Yasui et al. reported that elevated blood neutrophil counts (≥3,944/µL) in patients with severe asthma were independently associated with uncontrolled disease (OR: 4.05, 95% CI: 1.73–9.44) (25). When the analysis was restricted to patients receiving biologic therapy, the odds ratio for blood neutrophils ≥3,944/µL increased (OR: 6.16, 95% CI: 1.59–23.8). In our cohort, patients with both mixed and neutrophilic inflammatory patterns had blood neutrophil levels above the cut-off proposed by Yasui et al., and neutrophilic patients, in particular, exhibited a reduced response to biologic therapy compared with eosinophilic patients. Consistent with this study, our neutrophilic patients showed no significant changes in blood neutrophil counts over one year of follow-up, nor did they exhibit significant changes in sputum neutrophil levels.

It was recently reported that patients with a T2-low phenotype are less likely to achieve asthma remission than those with a T2-high phenotype (26). Consistently, our neutrophilic patients showed less improvement than eosinophilic patients after one year of treatment. Reduction of elevated blood eosinophil counts and/or FeNO levels and/or atopic status is undoubtedly important for disease stability, as demonstrated by the improvement in ACT and ACQ scores and by reduction of exacerbation rate. However, although most of our neutrophilic patients exhibited increased T2 biomarkers, persistent airway neutrophilic inflammation may explain the attenuated improvement in lung function observed following biologic therapy. Elevated total cell counts and neutrophil percentages in induced sputum could serve as biomarkers to identify patients who may benefit from azithromycin, a promising option for achieving clinical remission in the absence of T2 biomarkers (27). In addition, bacterial colonization and reduced lower airway microbial diversity with dysbiosis have been observed in patients with T2-low asthma (28), further supporting the potential use of azithromycin in this population. In our cohort, patients with a neutrophilic pattern exhibited higher total sputum cell counts at baseline, with no reduction after 12 months of biologic therapy. Treatment with azithromycin may represent a potential strategy to reduce the high sputum cell burden.

In the ATLANTIS cohort, the authors demonstrated that assessing blood and sputum neutrophilia in asthmatic patients is not useful for clinical phenotyping (29). However, according to our findings and those of others (26), identifying local neutrophilic inflammation may help predict the response to biologic therapy. Patients with severe asthma who showed a partial or no response to mepolizumab or benralizumab had elevated baseline sputum neutrophil percentages (30). In a small cohort of patients with severe asthma treated with benralizumab, elevated sputum IL-6 and IL-8 levels appeared to predict non-remission after one year of follow-up. These biomarkers showed a stronger positive correlation with both sputum neutrophils and macrophages, suggesting that the presence of T1-driven inflammation is associated with reduced clinical remission after one year (31). In a subgroup of patients with T2-high severe asthma, persistent elevation of blood neutrophils after biologic treatment was associated with poor asthma control and an increased risk of exacerbations (32). Our findings are consistent with these results, indicating that neutrophilic airway inflammation—even in the presence of elevated blood eosinophils or high FeNO levels—is associated with worse outcomes in patients treated with biologics.

It has also been reported that patients with severe asthma may experience an increase in exacerbations after 12–18 months of initially favorable response to biologic therapy. Several mechanisms have been proposed to explain this secondary loss of response, including the development of autoimmune processes, an increased susceptibility to viral and/or bacterial infections, and a shift in local airway inflammation from a T2-high to a T2-low phenotype (21). The evaluation of sputum cellular and molecular biomarkers may therefore be useful for better characterizing these patients and guiding adjustments in biologic therapy.

To the best of our knowledge, few studies have evaluated the impact of neutrophilic airway inflammation, as detected by induced sputum, on the outcomes of biologic therapy in patients with severe asthma. In our cohort, patients with severe asthma and evidence of airway neutrophilic inflammation showed reductions in exacerbation rates, symptom scores, and blood inflammatory biomarkers after at least one year of treatment; however, they maintained their neutrophilic airway inflammation and did not exhibit significant improvement in lung function or reduction in OCS use. Notably, most of these patients also presented with T2 biomarkers, such as elevated blood eosinophils, specific IgE, and/or high FeNO levels. Sputum analysis allowed direct assessment of bronchial inflammation, enabling a more precise characterization of these patients.

The limitations of our study include its monocentric, retrospective design and the relatively small number of subjects evaluated. Nevertheless, it highlights the potential value of sputum analysis for better characterizing and monitoring these patients, independently of the presence of other high T2 biomarkers. Further studies are needed to confirm our data.

In conclusion, the characterization of airway inflammation in patients with severe asthma treated with biologics makes it possible to identify those with neutrophilic inflammation—even in the presence of other T2-high biomarkers—who should be carefully monitored, particularly with regard to lung function.

## Data Availability

The raw data supporting the conclusions of this article will be made available by the authors, without undue reservation.

## Supplementary material

Supplementary Table 1 Change from baseline (Δ 6-0 and 12-0) of variables in patients divided according to their inflammatory phenotype at baseline.
