# Supplementary material for: Impact of sputum neutrophilia on the efficacy of biologics in severe asthma
Source: Front Allergy. 2026 Apr 8;7:1789783. doi: 10.3389/falgy.2026.1789783 (PMC13099925; doi:10.3389/falgy.2026.1789783)
Supplement: Supplementary Table 1 — Change from baseline (Δ 6-0 and 12-0) of variables in patients divided according to their inflammatory phenotype at baseline. [file Table1.pdf]

Supplementary Table 1S Change from baseline (Δ 6-0 and 12-0) of variables in patients divided according to their inflammatory phenotype at baseline

Variables	Eos patients	Mixed patients	Neutro patients	p value	Eos vs Mixed	Eos vs Neutro	Mixed vs Neutro
Δ T6-T0 ACQ	0.00 (-0.58-0.17)	0.00 (-1.00-0.00)	0.00 (0.00-0.82)	0.0508			
Δ T6-T0 ACT	2.00 (0.00-5.00)	3.00 (0.75-5.00)	3.00 (-0.50-6.50)	0.8649			
Δ T6-T0 FeNO	-18,00 (-50,50 - -3,50)	-16.00 (-91.50- 31.00)	0.00 (-8.00-3.00)	0.0627			
Δ T6-T0 BEC	-369.29 (-658.47- -51.04)	-475.33 (-743.21- -176.18)	-124.58 (-398.24- -40.81)	0.1865			
Δ T6-T0 FEV1	0.26 (0.00-0.66)	0.15 (0.00-0.40)	0.06 (-0.87-0.20)	0.0640			
Δ T6-T0 FVC	0.26 (-0.04-0.54)	0.15 (0.00-0.41)	0.10 (0.00-0.19)	0.3298			
Δ T12-T0 ACQ	0.00 (-0.83-0.17)	0.00 (-1.00-0.17)	0.17 (0.00-1.17)	**0.0174**	0.5620	0.0090	0.0130
Δ T12-T0 ACT	0.25 (0.00-6.00)	1.00 (0.00-4.50)	4.50 (0.25-6.75)	0.5549			
Δ T12-T0 FeNO	-13,00 (-69.00- 10.00)	-11.00 (-49.50-20.50	2.00 (-29.00-13.00)	0.7802			
Δ T12-T0 BEC	-384.50 (-612.12- -12.4.36)	-435.50 (-707.87- -143.51)	-203.78 (-436.76- -30.11)	0.2284			
Δ T12-T0 FEV1	0.35 (0.00-0.59)	0.27 (0.03-0.41)	-0.06 (-0.33-0.08)	**0.0231**	1.0000	**0.0180**	0,1650
Δ T12-T0 FVC	0.25 (-0.09-0.50)	0.19 (-0.13-0.43)	-0.11 (-0.41-0.40)	0.1683			

ACQ= Asthma Control Questionnaire; ACT= Asthma Control Test; FeNO= fractional exhaled nitric oxide; BEC= blood eosinophil count; FEV_1= Forced Expiratory Volume in 1 Second; FVC= Forced Vital Capacity. Statistically significant differences are shown in bold.
